# Total Anomalous Pulmonary Venous Connection in Mother and Son with a Central 22q11.2 Microdeletion

**DOI:** 10.1155/2021/5539855

**Published:** 2021-06-10

**Authors:** Signe Faurschou, Dorte L. Lildballe, Lisa L. Maroun, Morten Helvind, Maria Rasmussen

**Affiliations:** ^1^Department of Clinical Genetics, Lillebaelt Hospital, Vejle Hospital, University Hospital of Southern Denmark, Vejle, Denmark; ^2^Department of Pathology, Rigshospitalet, University Hospital of Copenhagen, Copenhagen, Denmark; ^3^Department of Cardiothoracic Surgery, Rigshospitalet, University Hospital of Copenhagen, Copenhagen, Denmark; ^4^Department of Regional Health Research, University of Southern Denmark, Odense, Denmark

## Abstract

In this clinical report, we describe a male infant and his mother, who had similar congenital heart defects. They were both diagnosed neonatally with total anomalous pulmonary venous connection (TAPVC) in combination with other heart defects. Neither of the two had any other organ malformations or dysmorphic facial features. SNP-array identified a central 22q11.2 microdeletion in the male infant and his mother as well as in the maternal grandmother and maternal aunt. The mother and the maternal aunt additionally harbored a 15q11.2 BP1-BP2 microdeletion. The maternal grandmother was unaffected by heart disease. However, heart computed tomography scan of the maternal aunt revealed a quadricuspid aortic valve. Additionally, the maternal grandmother and the maternal aunt both had significant learning disabilities. Rarely, TAPVC has been described in patients with the common 22q11.2 microdeletions. However, to the best of our knowledge, TAPVC has not previously been reported in patients with this small central 22q11.2 microdeletion. Haploinsufficiency of *TBX1* was originally thought to be the main cause of the 22q11.2 microdeletion syndrome phenotype, but *TBX1* is not included in the atypical central 22q11.2 microdeletion. Previous reports have suggested an association between TAPVC and the 15q11.2 BP1-BP2 microdeletion. Our report does not support this association as the maternal aunt, who harbors both microdeletions, is unaffected by TAPVC, and the male infant affected by TAPVC does not harbor the 15q11.2 BP1-BP2 microdeletion. Our findings support that genes located in the central 22q11.2 region are important for heart development and that haploinsufficiency of these genes plays a crucial role in the development of the rare heart defect TAPVC.

## 1. Introduction

Chromosome 22q11.2 includes a number of regions with highly repetitive DNA sequences referred to as low copy repeats (LCRs). These sequences are named LCR22-A to LCR22-H and cause chromosomal instability as they predispose to nonhomologous recombinations that lead to frequent microdeletions and microduplications in this region.

**Figure 1:**
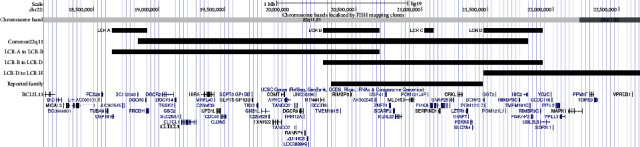
Illustration of the chromosome 22q11.2 region including the genes located in this region and the position of low copy repeats (LCRs). Also, the common and atypical 22q11.2 microdeletions are illustrated as well as the microdeletion identified in the reported family. The positions of the LCRs are depicted as reported in a previous study [1].The illustration is made in the UCSC genome browser (hg19 assembly) (http://genome.ucsc.edu).

The common 22q11.2 microdeletions have a size of approximately 3 Mb spanning from LCR22-A to LCR22-D (Figure 1) [2]. However, several other atypical, recurrent microdeletions in this region are identified. Previously, it has been suggested to categorize these atypical microdeletions into proximal, central, and distal microdeletions according to their specific locations: the proximal deletions spanning from LCR22-A to LCR22-B, the central deletions spanning from LCR22-B to LCR22-D, and the distal deletions extending beyond LCR22-D [3] (Figure 1).

The common 22q11.2 deletion syndrome is proposed to be one of the most common microdeletion syndromes [3, 4]. The incidence is estimated to 1 : 2,000–4,000 live births [5, 6]. However, the central microdeletions are rare and known to have reduced penetrance and variable expression. The phenotype may include congenital heart defects, dysmorphic features, and delay of development and growth [3, 7]. Rarely, anomalies of the palate and urogenital system, microcephaly, behavioral problems, and psychiatric disorders have been reported [3], whereas thymic dysfunction, endocrine disturbances, and gastrointestinal problems are primarily reported in patients with the common 3 Mb 22q11.2 microdeletion [8]. The most commonly reported heart defects in patients with the central 22q11.2 microdeletions are ventricular septal defect (VSD), atrial septal defect (ASD), and tetralogy of Fallot (TOF) [3]. Haploinsufficiency of three genes in the 22q11.2 region was primarily believed to be associated with the development of heart defects, *TBX1*, *CRKL*, and *MAPK1* [9, 10]. Among those, only *CRKL* is deleted in the central microdeletion (Figure 1).

Here, we present a mother and her son with total anomalous pulmonary venous connection (TAPVC). In TAPVC, venous blood from the pulmonary veins drains directly into the systemic veins or the coronary sinus. The incidence of TAPVC is 5.9–7.1 per 100,000 live births, and TAPVC represents only 1–5% of all congenital heart defects [11]. Familial cases of TAPVC are only rarely reported [12].

In this clinical report, we want to draw attention to the rare small central 22q11.2 microdeletion that seems to be associated with TAPVC in a mother and her son.

## 2. Case Presentation

The proband was a male infant born at gestational age 40 + 2 to nonconsanguineous Caucasian parents. The pregnancy was complicated by gestational diabetes, but all prenatal scans had been unremarkable. The birth weight was 3320 g.

Right after birth, the male infant needed treatment with continuous positive airway pressure (CPAP) and developed during the first night an increasing need for supplemental oxygen. Therefore, he was transferred to a larger hospital for more intensive care. An echocardiogram revealed a heart defect with TAPVC, where the pulmonary veins drained to the coronary sinus. The left atrium and left ventricle were hypoplastic, and a patent foramen ovale with right-to-left flow was seen. Also, he had a patent ductus arteriosus (PDA). Despite repeated surgeries, he died at the age of 12 days.

The autopsy revealed a heart with surgically corrected TAPVC, a hypoplastic left ventricle, and right coronary artery origin above the sinus of Valsalva. No other malformations were found, neither was he found to have dysmorphic features. Histology showed consequences of surgery and treatment with extracorporeal membrane oxygenation (ECMO), such as infarction in the myocardium, liver, and right adrenal gland.

His mother was also born with a congenital heart defect. She underwent surgery when she was five weeks old for TAPVC, PDA, atrial septal defect (ASD), and ventricular septal defect (VSD). Ultrasound examination of the kidneys was normal. She is 157 cm tall and has completed mainstream school. No other diseases were reported in her medical history.

Since the mother and son suffered from similar severe and rare congenital heart defects, the mother was referred for genetic counselling.

Trio-exome sequencing of the mother, father, and son did not identify any pathogenic or likely pathogenic variants in genes previously associated with congenital heart defects.

**Figure 2:**
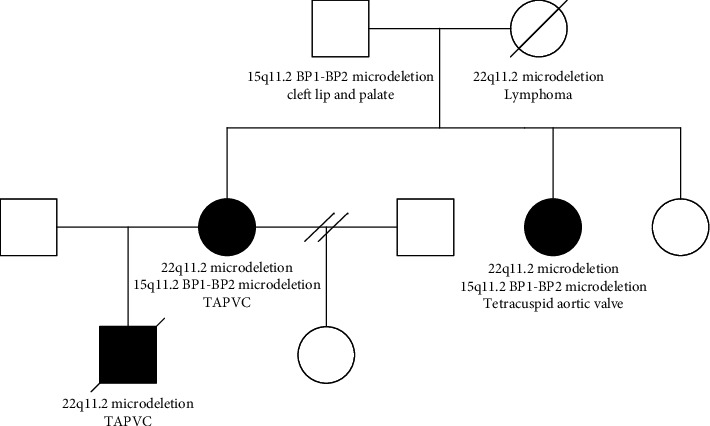
Pedigree of the family under study. Circles indicate females, squares indicate males, and black-filled symbols indicate family members affected by congenital heart defects.

Single-nucleotide polymorphism array (SNP-array) analysis of the son identified a 1.1 Mb 22q11.2 microdeletion (base pairs 20,306,993–21,462,353; genome build: hg19/GRCh37). This deleted region is referred to as LCR B-to-D. Subsequently, the same microdeletion was identified in the mother. Additionally, a 15q11.2 BP1-BP2 microdeletion (base pairs 20,591,187–23,222,284; genome build: hg19/GRCh37) was identified in the mother. SNP-array of the maternal grandfather, who was born with cleft lip and palate, did not identify the 22q11.2 microdeletion, but only the 15q11.2 BP1-BP2 microdeletion. The maternal grandmother had died of lymphoma. Therefore, DNA extracted from paraffin-embedded tissue was tested postmortem. SNP-array was not possible, but multiplex ligation-dependent probe amplification (MLPA) assay identified the identical 22q11.2 microdeletion in the maternal grandmother. She was not diagnosed with any heart disease, but she had significant learning disability and had attended special school. Lastly, SNP-array of the maternal aunt identified the 22q11.2 microdeletion as well as the 15q11.2 BP1-BP2 microdeletion. The maternal aunt is 155 cm tall. Echocardiography and ultrasound examination of the kidneys were normal, but heart computed tomography scan revealed a quadricuspid aortic valve. She has significant learning disability and has attended the same special school as her mother. Also, several minor dysmorphic facial features were noted, see pedigree (Figure 2).

Written informed consent for publication was obtained from the mother, the maternal aunt, and the maternal grandfather.

## 3. Discussion

In this clinical report, we report a male infant and his mother, who had similar severe, isolated heart defects and were both found to have a small central 22q11.2 microdeletion. For this reason, we assume that the microdeletion plays a crucial role in the development of this specific congenital heart defect, TAPVC. To our knowledge, the small central microdeletion has not previously been associated with TAPVC. Congenital heart defects are previously reported in patients with the central 22q11.2 microdeletion, but they are usually less severe.

In the DECIPHER database [13], we identified 75 additional patients with deletions smaller than 1.5 Mb that overlapped with the deletion identified in the proband and his mother. Among the 75 patients, 11 had a reported heart diagnosis. Neither of these 11 patients had TAPVC. One study found that 17% of 46 patients with central B-to-D or C-to-D microdeletions, that included *CRKL* but not *TBX1*, had congenital heart defects [3]. Subsequently, another study reviewed 101 patients with central deletions and found that 20% of the patients had congenital heart defects [7].

One study has described an association between 22q11.2 copy number variants and anomalous pulmonary venous connection (APVC) [11]. Using MLPA, they found that 1 in 86 patients with APVC had a 22q11.2 microdeletion. This one patient had TAPVC and VSD, however, the deletion was larger than the one described in our study, approximately 2 Mb, and included *TBX1* as well as *CRKL*.

The importance of the size of the 22q11.2 microdeletions has been studied previously. It has repeatedly been found that the size of the microdeletions seems not to correlate with the clinical symptoms [4, 10, 14]. This is well in line with the findings presented in this report, in which an identical microdeletion seems to have caused a severe heart defect in the mother and son, a minor heart defect in the maternal aunt, and apparently no heart defect in the maternal grandmother. The underlying causes for this intrafamilial disease variability are unknown but probably include the presence of genetic modifiers outside the 22q11.2 region as well as environmental factors.

The central 22q11.2 microdeletion overlaps 40 HGNC genes of which 16 genes are OMIM genes. None of the individual genes are known to cause disease in humans when deleted in heterozygous form. However, particularly the *CRKL* gene has previously been proposed to play an important role in the development of congenital heart defects, either independently or through modification of *TBX1* expression [9, 10]. Also, mouse models with altered *CRKL* expression have supported that *CRKL* has a role in the development of congenital heart defects [15–17].

Motahari et al. have suggested that besides from the *CRKL* gene, the *PI4KA* and *LZTR1* genes, which are also included in this central 22q11.2 microdeletion, are implicated in the heart phenotype [18]. In zebrafish *PI4KA* loss-of-function disrupts the brain, heart, and trunk development [18]. However, the implication of *PI4KA* in the heart phenotype has not been supported in mammalian model organisms, but multiple reports implicate *PI4KA* in the neuropsychiatric phenotype [18, 19], and biallelic pathogenic variants in *PI4KA* are known to cause perisylvian polymicrogyria, cerebellar hypoplasia, and arthrogryposis in humans [20].

Heterozygous and biallelic, pathogenic variants in *LZTR1* cause Noonan syndrome. Heart malformations are common in Noonan syndrome, particularly pulmonary valve stenosis, septal defects, and coarctation of the aorta. Steklov et al. demonstrated that *LZTR1* haploinsufficiency in mice recapitulates the Noonan syndrome phenotype including various heart malformations [21].

Overall, *CRKL* and *LZTR1* seem to be the strongest candidate genes in the central 22q11.2 region implicated in the heart phenotype.

Other genes outside the 22q11.2 region have also been proposed to be associated with the development of TAPVC. One study investigated 178 TAPVC patients by next-generation sequencing and identified three candidate genes *SNAI1*, *HMGA2*, and *VAV2* that seemed to be important for the development of TAPVC [22]. However, the association of these genes with TAPVC has so far not been confirmed in other studies, and no rare variants in these genes were identified in the mother and her son by trio-exome sequencing.

Another study investigated 231 TAPVC patients and found 13 patients with a 15q11.2 microdeletion, whereas the 15q11.2 microdeletion was not found in 200 controls [23]. In a previous case report, a 15q11.2 BP1-BP2 microdeletion has been reported in two of three siblings with TAPVC and in a healthy father. The third sibling was not available for genetic analysis [24]. However, the association of the 15q11.2 BP1-BP2 microdeletion and development of congenital heart defects is uncertain. A recently published meta-analysis found no increased risk of congenital heart defects in patients with 15q11.2 BP1-BP2 microdeletions [25].

This clinical report supports the previous assumption that *CRKL* and *LZTR1* are important for heart development, and with this case, we suggest that *CRKL* and *LZTR1* haploinsufficiency may play a crucial role in the development of TAPVC, although the phenotype may vary even within the same family.
